# Clinical and genetic characteristics of children with acute lymphoblastic leukemia and Li–Fraumeni syndrome

**DOI:** 10.1038/s41375-021-01163-y

**Published:** 2021-02-12

**Authors:** Greta Winter, Renate Kirschner-Schwabe, Stefanie Groeneveld-Krentz, Gabriele Escherich, Anja Möricke, Arend von Stackelberg, Martin Stanulla, Simon Bailey, Lisa Richter, Doris Steinemann, Tim Ripperger, Adela Escudero, Roula Farah, Olli Lohi, Karin Wadt, Marjolijn Jongmans, Nienke van Engelen, Cornelia Eckert, Christian Peter Kratz

**Affiliations:** 1 Pediatric Hematology and Oncology, Hannover Medical School, Hannover, Germany; 2 Department of Pediatric Oncology and Hematology, Charité—University Medical Center Berlin, Berlin, Germany; 3 German Cancer Consortium (DKTK) and German Cancer Research Center (DKFZ), Heidelberg, Germany; 4 Pediatric Hematology and Oncology, University Medical Centre Hamburg-Eppendorf, Hamburg, Germany; 5 Department of Pediatrics, Christian-Albrechts-University Kiel, Kiel, Germany; 6 Great North Children’s Hospital and Newcastle University, Newcastle upon Tyne, UK; 7 Pediatric Hematology and Oncology, University Hospital Essen, Essen, Germany; 8 Department of Human Genetics, Hannover Medical School, Hannover, Germany; 9 Translational Research in Pediatric Oncology, Hematopoietic Stem Cell Transplantation and Cell Therapy, Institute of Medical and Molecular Genetics, Hospital La Paz Institute for Health Research (IdiPAZ), Madrid, Spain; 10 Department of Pediatrics, Lebanese American University Medical Center-Rizk Hospital, Beirut, Lebanon; 11 Faculty of Medicine and Health Technology, Tampere University and Tays Cancer Centre, Tampere University Hospital, Tampere, Finland; 12 Department of Clinical Genetics, Copenhagen University Hospital, Rigshospitalet, Copenhagen, Denmark; 13 Department of Genetics, University Medical Center Utrecht, Utrecht, The Netherlands; 14 Princess Máxima Center for Pediatric Oncology, Utrecht, The Netherlands

**Keywords:** Acute lymphocytic leukaemia, Genetics research, Risk factors

## To the Editor:

Approximately 10% of cases of childhood cancer arise in the context of a cancer predisposition syndrome (CPS) [1]. Among the rare CPS, Li–Fraumeni syndrome (LFS, MIM#151623) is relatively common and estimated to account for >1% of cases of childhood cancer [2]. LFS is a dominantly inherited condition caused by pathogenic germline variants in the *TP53* tumor suppressor gene [3, 4]. Children with LFS are predisposed to a range of neoplasms such as osteosarcoma, adrenocortical carcinoma, medulloblastoma, choroid plexus carcinoma, anaplastic rhabdomyosarcoma, and (frequently hypodiploid) acute lymphoblastic leukemia (LFS-ALL) [5, 6]. Notably, somatic mutations and germline variants of *TP53* are enriched at relapse and are associated with poor prognosis in relapsed childhood ALL [7]. A recent study involving 3801 children with ALL showed that LFS-ALL accounts for less than 1% of ALL cases [8] and was associated with inferior event-free survival as well as overall survival, and higher risk of second malignant neoplasms (SMN) [8]. Nevertheless, the clinical and genetic characteristics of LFS-ALL are poorly studied. Therefore, we conducted a retrospective cohort study on 18 children with LFS-ALL to further delineate the characteristics of this rare association.

All 18 ALL patients were identified as having LFS based on the presence of a (likely) pathogenic *TP53* germline variant (see also Supplementary Table 1). Six patients were identified in a cohort of patients with relapsed ALL among 564 analyzed patients with relapsed ALL registered in the multicentre randomized trial *ALL-REZ BFM 2002*. At the time of initial ALL diagnosis, all six patients were enrolled in one of the following multicentre randomized trials for the treatment of newly diagnosed ALL: *ALL-BFM 95*, *ALL-BFM 2000*, *COALL 06-97*, or *COALL 07-03*. Clinical and genetic data were collected from the study centers in Kiel, Hamburg, and Berlin and provided by associated research laboratories, respectively. Twelve additional cases were recruited through the international BFM study group. Here, the diagnosis of LFS was established at various timepoints during the clinical course whenever the treating physicians suspected the diagnosis of LFS based on clinical findings (e.g., hypodiploid karyotype or cancer history). Relevant clinical and genetic data on all 18 LFS-ALL patients were collected and evaluated retrospectively. The study was approved by the ethical review board at Hannover Medical School.

**Fig. 1:**
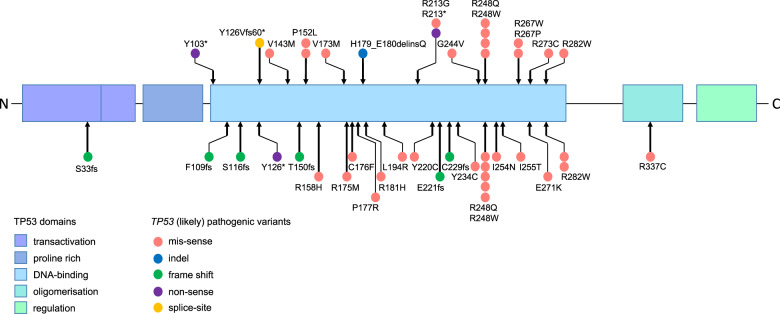
Pathogenic/likely pathogenic TP53 germline variants in patients with LFS-ALL. The variants described as pathogenic or likely pathogenic are marked with a red circle (missense), blue circle (indel), green circle (frame shift), purple circle (nonsense), and yellow circle (splice-site). The lower part of the figure shows the variants described in [8] (color figure online).

Pathogenic/likely pathogenic *TP53* variants observed in this study are depicted in Fig. 1. All patients carried variants located within the DNA-binding domain. One individual harbored a variant that is predicted to result in aberrant splicing, probably leading to skipping of exon 5 which will induce a frame shift and premature stop within the DNA-binding domain. Six patients carried pathogenic germline *TP53* variants p.R248Q (*n* = 3), p.R248W, p.R273C, or p.R282W, which represent known somatic and germline hotspot alterations in *TP53* [9]. One specific amino acid was significantly more frequently affected than expected. Four variants altered the arginine at codon 248 (Arg248) relevant for DNA contact. Together with a previous study [8] that showed that pathogenic/likely pathogenic variants altered the p53 protein at codon Arg248 in 5 out of 26 LFS-ALL cases, this represents a clustering of these variants in 9/44 (~20%) patients with LFS-ALL. This finding suggests that LFS patients with a variant affecting this position may have a particularly high ALL risk. The observation that Arg248 variants represent a hotspot in LFS-ALL highlights the importance of this confirmatory study. Codon Arg248 variants are among the most common germline variants in LFS and the high cancer risk is not restricted to ALL.

Clinical features were in agreement with reported findings [2]. All patients had B-cell precursor ALL. The mean age at the initial onset of ALL was 10.9 years and was significantly higher than the mean age at ALL diagnosis in children with sporadic disease (5.7 years, *P* < 0.001, for comparison, data from a cohort of 4664 patients with precursor B-ALL included in the multi-center trials ALL-BFM 95 and ALL-BFM 2000 were used). Six patients developed SMNs. Five out of 18 patients died within the documented observation period (median age at last follow-up: 14 years, range 4–34 years). Two patients died due to therapy-related multi-organ failure (patients 2 and 3) and three individuals deceased due to relapsed ALL (patients 5, 6, and 11). Nine patients suffered from relapsed ALL, however, this high number may be due to selection bias because six patients were identified though the relapsed ALL study center. The analysis of common leukemia fusion genes showed a *BCR/ABL* and *ETV6/RUNX1* rearrangement in two distinct patients. Eight patients had a hypodiploid or low hypodiploid karyotype at time of initial diagnosis. A hyperdiploid karyotype was documented in another three children, potentially due to masked hypodiploidy [10, 11]. Clinical features are summarized in Table 1 and Supplementary Table 2. Notably, patient 16 in Table 1 had LFS and Down syndrome. The high mean age of patients with LFS-ALL may reflect a unique underlying biology of LFS-ALL. This is also supported by the observation that, while gene fusions are a common feature of childhood ALL, only two translocations were detected in this cohort (patients 8 and 15), indicating that the presence of such rearrangements is rare in LFS-ALL but, importantly, cannot exclude an underlying diagnosis of LFS. As described previously and confirmed in this cohort, LFS-ALL is often associated with a hypodiploid karyotype [5].

**Table 1:** Overview of selected clinical features of patients with LFS-ALL.

Pat. ID	Pub. ID	Age in years at first diagnosis	ALL type	Sex	Cancer in family	Ploidy at initial diagnosis	Ploidy at relapse	*TP53* germline variant*	Leukemia fusion genes	Relapses	Second malignant neoplasms (age in years)	HSCT/Indication	HSCT conditioning regimen	Outcome (age in years)
1	115	15	BCP	M	Yes	Diploid	Masked low hypodiploid	p.(R248Q)	No	1	–	1/b	TBI, Cy, ATG	Alive (29)
2	463	13	BCP	M	No	n.a.	Low hypodiploid	p.(R248Q)	No	1	ALL (13) → LGG (13)	–	–	Deceased (13)
3	474	12	BCP	F	No	n.a.	Masked low hypodiploid	p.(R248W)	n.a.	1	ALL (4) → MDS (21)	2/b,c	TBI, VP16, ATG Flu, TT, Melph, ATG	Deceased (24)
4	478	2	BCP	F	No	Diploid	Diploid	p.(R267W)	No	1	–	–	–	Alive (16)
5	301	13	BCP	F	Yes	Hypodiploid	Low hypodiploid	p.(R273C)	No	1	–	–	–	Deceased (14)
6	351	7	BCP	F	No	Pseudodiploid	Low hypodiploid	p.(V173M)	No	3	–	2/b,b	TBI, VP16, ATG TT, Flu, Treo, ATG	Deceased (13)
7	–	8	BCP	F	Yes	Low hypodiploid	n.a.	p.(R213*)	No	1	–	–	–	Alive (n.a.)
8	–	11	BCP	M	n.a.	Diploid	–	p.(R282W)	*BCR/ABL*	–	–	–	–	alive (13)
9	–	8	BCP	F	Yes	Hypodiploid	–	p.(V143M)	no	–	–	1/a	Flu, Treo, TT	Alive (n.a.)
10	–	14	BCP	M	No	Masked hypodiploid	–	p.(Y126Vfs60*)	no	–	–	–	–	Alive (n.a.)
11	–	9	BCP	F	Yes	Diploid	Hyperdiploid	p.(R248Q)	No	1	NBL (0,5) → ALL (9)	–	–	Deceased (12)
12	–	15	BCP	M	Yes	Hypodiploid	–	p.(P152L)	No	–	–	–	–	Alive (n.a.)
13	–	9	BCP	F	Yes	Hyperdiploid	–	p.(Y103*)	No	–	ALL (9) → BC (34)	–	–	Alive (34)
14	–	14	BCP	M	Yes	Masked hypodiploid	–	p.(P152L)	No	–	–	1/a	TBI, VP16, ATG	Alive (n.a.)
15	–	2	BCP	M	Yes	Hyperdiploid	–	p.(R213G)	*ETV6/RUNX1*	–	–	–	–	Alive (4)
16	–	12	BCP	F	No	Low hypodiploid	–	p.(R267P)	No	–	ACC (1) → ALL (12)	–	–	Alive (12)
17	–	18	BCP	M	Yes	Hyperdiploid	–	p.(G244V)	No	–	OS (14) → ALL (18)	–	–	Alive (18)
18	675	14	BCP	M	Yes	Masked low hypodiploid	Masked low hypodiploid	p.(H179_E180delinsQ)	No	1	–	1/b	Flu, Bu, ATG, TT	Alive (18)

*Pat. ID* patient ID, *Pub. ID* already existing publication ID, *BCP* B-cell precursor, LGG low grade glioma, *MDS* myelodysplastic syndrome, *NBL* neuroblastoma, *BC* breast cancer, *ACC* adrenal cortical carcinoma, *OS* osteosarcoma, *HSCT* hematopoietic stem cell transplantation, *HSCT indication: a*. part of initial ALL treatment*, b*. part of ALL-relapse treatment, *c*. part of SMN treatment, *TBI* total body irradiation, *Cy* cyclophosphamide, *ATG* antithymocyte globulin, *VP16* etoposide, *Flu* fludarabine, *TT* thiotepa, *Melph* Melphalan, *Treo* treosulfan, *Bu* Busulfan, *n.a*. information not available.

*Refers to NM_000546.

To describe, for the first time, the range of therapy-related toxicities in individuals with LFS-ALL, we collected information on the occurrence of a range of acute toxic effects [12] (Supplementary Table 3). Notably, hypercalcemia, which is an extremely rare complication in childhood ALL [13, 14], occurred in one patient (total blood calcium concentration of 3.89 mmol/L). While the number of patients is too small to draw definite conclusions our data do not suggest that LFS patients have an acute toxicity profile that differs from the toxicities observed in patients with sporadic ALL.

Little is known about how LFS-ALL patients tolerate hematopoietic stem cell transplantation (HSCT) procedures. Therefore, we collected data on the conditioning regimens and transplant-related toxicities. Eight HSCT were performed in six patients.

The patients were transplanted during their initial ALL therapy (*n* = 2), due to ALL relapse (*n* = 5), or because of myelodysplastic syndrome (MDS) following ALL treatment (*n* = 1). Two patients required two HSCT procedures due to a second ALL relapse (patient 6) and due to MDS (patient 3) (Table 1 and Supplementary Table 4). While the numbers are too small to draw definite conclusions, LFS is not a contraindication for HSCT. Nevertheless, given the poorer outcome of patients with LFS-ALL [8] as well as the notion that genotoxic regimens are not ideal in the context of a germline defect in *TP53*, alternative treatment arms (e.g., employing immunotherapeutic elements) should be discussed by ALL trial groups. Until new LFS-ALL trial strategies have been developed, we do not advice against the use of standard treatment protocols, including HSCT, but cancer surveillance should be offered to all children with LFS-ALL [6].

In conclusion, our study validates the association between *TP53* germline variants and ALL. Children with LFS-ALL are significantly older than children with sporadic ALL reflecting a different underlying biology. In agreement with this notion, typical ALL translocations are rare in LFS-ALL but do not exclude the diagnosis of LFS. Pathogenic *TP53* germline variants affecting codon Arg248 may be associated with a particularly high ALL risk. Although no unexpected side effects were observed, it is conceivable that LFS-ALL patients could benefit from less genotoxic treatment strategies [15] in order to improve outcome and to avoid SMNs. Future studies are required to improve the prognosis of patients with LFS-ALL.

## Supplementary information

Supplementary Table 1

Supplementary Table 2

Supplementary Table 3

Supplementary Table 4
