# Supplementary material for: Clinical and genetic characteristics of children with acute lymphoblastic leukemia and Li–Fraumeni syndrome
Source: Leukemia. 2021 Feb 12;35(5):1475–9. doi: 10.1038/s41375-021-01163-y (PMC8102191; doi:10.1038/s41375-021-01163-y)
Supplement: Supplementary file 1 — Supplementary Table 1 [file 41375_2021_1163_MOESM1_ESM.docx]

**Supplementary Table 1** *TP53* germline variants

The table includes 2 variants (italic) that were classified as VUS. These are not part of the analysis.

| **variant^#^** | **GRCh38** | **IARC** | **ClinVar** | **dbSNP** | **class** | **criteria** |
| --- | --- | --- | --- | --- | --- | --- |
| c.309C>G p.(Y103*) | g.7676060G>C | 1046 | 822878 |  | pathogenic | PVS1 (NMD predicted); PM2_supporting, PS4_supporting (1 point) |
| *c.329G>A p.(R110H)* | *g.7676040C>T* | *1132* | *127808* | *rs11540654* | *likely benign* | *VCEP^TP53^* |
| c.427G>A p.(V143M) | g.7675185C>T | 1586 | 142657 | rs587782620 | likely pathogenic | PS3 (Kato, Giacomelli), PM1 (cancer hotspot), PM2_supporting; cave: conflicting entries in ClinVar |
| c.455C>T p.(P152L) | g.7675157G>A | 1741 | 142766 | rs587782705 | pathogenic | PS3 (Kato, Giacomelli), PM1 (cancer hotspot), PP3_moderate (C65, 0.5581), PS4_moderate (2 point) |
| c.517G>A p.(V173M) | g.7675095C>T | 2094 | 233951 | rs876660754 | likely pathogenic | PS3 (Kato, Giacomelli), PM1 (cancer hotspot), PP3_supporting (C15, 0.5927) PM2_supporting |
| c.537_539del p.(H179_E180delinsQ) | g.7675073_7675075del | – | – | – | likely pathogenic | PVS1_strong, PM1 (cancer hotspot), PM2_supporting, PS4_supporting |
| c.559+1G>T p.?. | g.7675052C>A | 5727 |  | rs1131691042 | pathogenic | PVS1^$^; PS2_moderate (1 point), PM2_supporting |
| c.637C>G p.(R213G) | g.7674894G>C | 2704 | 376651 | rs397516436 | likely pathogenic | PS3 (Kato, Giacomelli), PM1 (cancer hotspot), PP3_moderate (C65, 0.5216), PM2_supporting |
| c.637C>T p.(R213*) | g.7674894G>A | 2705 | 43590 | rs397516436 | pathogenic | PVS1 (NMD predicted); PS4_moderate (2 points), PM2_supporting |
| c.731G>T p.(G244V) | g.7674232C>A | 3222 | 376601 | rs985033810 | likely pathogenic | PS3 (Kato et al. and Kotler et al.), PP3_moderate (C65, 0.5970), PS2_moderate (2 points), PM2_supporting |
| c.742C>T p.(R248W) | g.7674221G>A | 3294 | 12347 | rs121912651 | likely pathogenic | PS3 (Kato, Giacomelli), PM1 (general hotspot), PP3_moderate, rare freq in gnomAD |
| c.743G>A p.(R248Q) | g.7674220C>T | 3297 | 12356 | rs11540652 | likely pathogenic | PS3 (Kato, Giacomelli), PM1 (general hotspot), PS2_moderate (1.5points), PP3_supporting (C35, 0.4738) |
| c.799C>T p.(R267W) | g.7673821G>A | 3633 | 141764 | rs587780075 | likely pathogenic | PS3 (Kato, Kotler), PM1 (cancer hotspot), PP3_moderate (C65, 0.5424), PM2_supporting (absent gnomAD) |
| c.800G>C p.(R267P) | g.7673820C>G | 3635 | 428867 | rs587780075 | pathogenic | PS3 (Kato Giacomelli), PM1 (cancer hotspot), PP3_moderate (C65, 0.5901), PS2_moderate (1.5 points), PM2_supporting |
| c.817C>T p.(R273C) | g.7673803G>A | 3730 | 43594 | rs121913343 | likely pathogenic | PS3 (Kato Giacomelli), PM1 (cancer hotspot), PP3_moderate (C65, 0.5537) |
| c.844C>T p.(R282W) | g.7673776G>A | 3879 | 12364 | rs28934574 | likely pathogenic | PS3 (Kato Giacomelli), PM1 (general hotspot), PP3_moderate (C65, 0.5418, PS4_moderate (2 points) |
| *c.892G>A p.(E298K)* | *g.7673728C>T* | *4119* | *141483* | *rs201744589* | *likely benign* | *BS3 (Kato Giacomelli), BP4 (C0, –0.1989)* |

#, variant description follows the recommendation of HGVS and refers to NM_000546; IARC, mutation ID in the international agency of research in cancer TP53 database, <https://p53.iarc.fr/TP53GeneVariations.aspx>, accessed 2020/09/01 [1]; ClinVar, https://www.ncbi.nlm.nih.gov/clinvar; dbSNP, https://www.ncbi.nlm.nih.gov/snp/; class, variant classification following ClinGen *TP53* expert panel specification to the ACMG/AMP variant interpretation guidelines version 1 based on Richards et al. [2]; criteria, applied specified ACMG/AMP criteria including respective details: (i) Kato, Giacomelli, and Kotler refer to the following publications [3], [4], and [5], respectively; (ii) scores provided for BP4 and PP3 refer, if not otherwise specified, to the aGVGD and BayesDel scores recommended by the ClinGen expert panels; REVEL, prediction tool for missense variants integrating 13 individual tools [6]; PVS1 criteria is applied with respect to the variant interpretation recommendations [7]; VCEP*^TP53^*, this variant was classified by the ClinGen *TP53* Variant Curation Expert Panel; ^$^, substitution c.559+1G>T is affecting the splice donor site at the end of exon 5; MAXEntScan predicts loss of the donor splice site (-100%), no cryptic splice site is detected; thus, exon skipping is assumed that would lead to the here assumed frame shift and premature termination that will theoretically lead to non-sense mediated decay.

**References**

1. Bouaoun L, Sonkin D, Ardin M, Hollstein M, Byrnes G, Zavadil J, et al. TP53 Variations in Human Cancers: New Lessons from the IARC TP53 Database and Genomics Data. Human Mutation. 2016;37:865-76.

2. Richards S, Aziz N, Bale S, Bick D, Das S, Gastier-Foster J, et al. Standards and guidelines for the interpretation of sequence variants: a joint consensus recommendation of the American College of Medical Genetics and Genomics and the Association for Molecular Pathology. Genet Med. 2015;17:405-24.

3. Kato S, Han SY, Liu W, Otsuka K, Shibata H, Kanamaru R, et al. Understanding the function-structure and function-mutation relationships of p53 tumor suppressor protein by high-resolution missense mutation analysis. Proc Natl Acad Sci U S A. 2003;100:8424-9.

4. Giacomelli AO, Yang X, Lintner RE, McFarland JM, Duby M, Kim J, et al. Mutational processes shape the landscape of TP53 mutations in human cancer. Nat Genet. 2018;50:1381-7.

5. Kotler E, Shani O, Goldfeld G, Lotan-Pompan M, Tarcic O, Gershoni A, et al. A Systematic p53 Mutation Library Links Differential Functional Impact to Cancer Mutation Pattern and Evolutionary Conservation. Mol Cell. 2018;71:178-90 e8.

6. Ioannidis NM, Rothstein JH, Pejaver V, Middha S, McDonnell SK, Baheti S, et al. REVEL: An Ensemble Method for Predicting the Pathogenicity of Rare Missense Variants. Am J Hum Genet. 2016;99:877-85.

7. Abou Tayoun AN, Pesaran T, DiStefano MT, Oza A, Rehm HL, Biesecker LG, et al. Recommendations for interpreting the loss of function PVS1 ACMG/AMP variant criterion. Hum Mutat. 2018;39:1517-24.
