# Supplementary material for: Clinical and genetic characteristics of children with acute lymphoblastic leukemia and Li–Fraumeni syndrome
Source: Leukemia. 2021 Feb 12;35(5):1475–9. doi: 10.1038/s41375-021-01163-y (PMC8102191; doi:10.1038/s41375-021-01163-y)
Supplement: Supplementary file 2 — Supplementary Table 2 [file 41375_2021_1163_MOESM2_ESM.docx]

**Supplementary Table 2** Clinical features of 18 patients with LFS-ALL

| **Characteristic** | |
| --- | --- |
| **Mean age in years at first diagnosis** (n=18) | 10.89 (range: 2-18) (*P* <.001) |
| **Sex** (n=18) | |
| female | 9 |
| male | 9 |
| **Cancer history in family members** (n=17) | |
| positive | 11 |
| negative | 6 |
| **De novo vs. inherited** (n=6) | |
| *de-novo* mutation | 3 |
| *TP53* germline variant known in family | 3 |
| **ALL relapse** (n=18) | |
| at least one* | 9 |
| none | 9 |
| **Second malignant neoplasm** (n=18) | |
| Yes | 6** |
| No | 12 |
| **Vital status at last follow-up** (n=18) | |
| deceased | 5 |
| alive | 13 |
| **Ploidy first ALL** (n=16) | |
| diploid | 4 |
| (masked) hypodiploid | 5 |
| (masked) low hypodiploid | 3 |
| hyperdiploid | 3 |
| pseudodiploid | 1 |
| **Ploidy first ALL relapse** (n=8) | |
| diploid | 1 |
| (masked) hypodiploid | 0 |
| (masked) low hypodiploid | 6 |
| hyperdiploid | 1 |
| **Leukemia fusion genes first ALL** (n=17) | |
| *BCR/ABL* | 1 |
| *MLL* | 0 |
| *ETV6/RUNX1* | 1 |
| **Initial leucocyte count ≥50.000/μl** | |
| at first diagnosis (n=18) | 0 |
| at first relapse (n=6) | 0 |
| **CNS-status >2** | |
| at first diagnosis (n=16) | 0 |
| at first relapse (n=5) | 0 |
| **Bone marrow blasts on day 15** (n=14) | |
| <5% at first diagnosis | 11 |
| ≥5% at first diagnosis | 3 |
| **≥10^-3^ MRD in week 4-5** | |
| at first diagnosis (n=11) | 5 |
| at first relapse (n=4) | 2 |

* This may be due to selection bias (*TP53* mutation testing was done in patients with relapsed ALL).

**In 3 patients ALL occurred as second malignant neoplasm.
