# Supplementary material for: Clinical and genetic characteristics of children with acute lymphoblastic leukemia and Li–Fraumeni syndrome
Source: Leukemia. 2021 Feb 12;35(5):1475–9. doi: 10.1038/s41375-021-01163-y (PMC8102191; doi:10.1038/s41375-021-01163-y)
Supplement: Supplementary file 3 — Supplementary Table 3 [file 41375_2021_1163_MOESM3_ESM.docx]

**Supplementary Table 3** Adverse treatment reactions observed in 18 patients with LFS-ALL

| **Adverse reaction** | **Patients** |
| --- | --- |
| Hypersensitivity to asparaginase (n=18)*  anaphylaxis with indication for interruption of asparaginase  and/or parenteral medication (e.g. antihistamines)  no data to severity | 7  3  4 |
| Osteonecrosis (n=18)*  confirmed by imaging and limiting activity of daily life  no data to severity | 2  1  1 |
| Posterior reversible encephalopathy syndrome (n=9)* | 1 |
| Seizures (n=18)*  brief generalised seizure | 1 |
| MTX-related stroke-like syndrome (n=18)*  suspicious finding by imaging and/or biopsy, new onset of severe neurological symptoms e.g. seizures, no other identifiable cause | 1 |
| Peripheral neuropathy (n=18)* | 5 |
| Arterial hypertension (n=18)* | 4 |
| Thromboembolism (n=18)*  deep vene thromboembolism and/or indication of systemic  anticoagulation | 2 |
| Hyperlipidemia (n=17)*  triglycerides/cholesterol upper normal limit | 1 |
| Asparaginase-associated pancreatitis (n=18)* | 1 |
| Depressed level of consciousness (n=18)* | 1 |
| HD-MTX-related severe Nephrotoxicity (n=18)* | 0 |
| Sinusoidal obstruction syndrome (n=18)* | 0 |
| Hypercalcemia ≥ 10.5 mg/dL** (n=18) | 1 |
| Severe mucositis (WHO III-IV°) (n=18) | 5 |

*These side effects are based on a consensus on acute toxic effects for childhood lymphoblastic leukemia [Schmiegelow et al.] and have been slightly modified.

**Hypercalcemia is reported for total blood calcium concentration.
