# Supplementary material for: Clinical and genetic characteristics of children with acute lymphoblastic leukemia and Li–Fraumeni syndrome
Source: Leukemia. 2021 Feb 12;35(5):1475–9. doi: 10.1038/s41375-021-01163-y (PMC8102191; doi:10.1038/s41375-021-01163-y)
Supplement: Supplementary file 4 — Supplementary Table 4 [file 41375_2021_1163_MOESM4_ESM.docx]

**Supplementary Table 4** Conditioning regimen, adverse reactions and outcome of 6 patients with LFS-ALL treated with hematopoietic stem cell transplantation (HSCT).

| **General details** | **Number** | | |
| --- | --- | --- | --- |
| patients treated with HSCT | 6 | | |
| performed HSCTs^*^ | 8 | | |
| **Conditioning regimen (for first HSCT)** (n=6) |  | | |
| total body irradiation | 4 | | |
| chemotherapy only | 2 | | |
| **Adverse reactions of eight HSCTs** |  | | |
| **Conditioning period** | | | |
| reaction to ATG (n=7) | 1 | | |
| fever (n=8) | 4 | | |
| arterial hypertension (n=8) | 4 | | |
| **Transplantation period** | | | |
| infection (n=8) | 5 | | |
| fever (n=8) | 6 | | |
| renal toxicity or hemorrhagic cystitis (n=8) | 3 | | |
| nausea, vomiting, gastrointestinal disorders (n=6) | 4 | | |
| severe mucositis (n=8) | 7 | | |
| neuropathia (n=8) | 3 | | |
| reversible cardiovascular disorders (n=8) | 1 | | |
| delayed leucocyte entgraftment (n=7) | 2 | | |
| maximum GVHD >II° (n=7) | 1 | | |
| **Outcome** (n=6) |  | | |
| deceased | 2 | | |
| alive | 4 | | |
| **Conditioning regimen with TBI (for first HSCT)** (n=4) | |  | |
| second malignant neoplasm after HSCT | 1 | | |
| relapse after HSCT | 1 | | |
| deceased | 2 | | |
| alive | 2 | | |
| **Conditioning regimen with chemotherapy only (for first HSCT)** (n=2) | | |  |
| second malignant neoplasm after HSCT | 0 | | |
| relapse after HSCT | 0 | | |
| deceased | 0 | | |
| alive | 2 | | |

^*^2 patients were transplanted twice due to myelodysplastic syndrome or second ALL relapse
